# The differences in cocoon and silk qualities among sex-related mulberry and silkworm feeding groups

**DOI:** 10.1371/journal.pone.0270021

**Published:** 2022-06-30

**Authors:** Chunlan Bu, Rui Zheng, Gaiqun Huang, Jianmei Wu, Gang Liu, Marion L. Donald, Tingfa Dong, Xiao Xu

**Affiliations:** 1 Key Laboratory of Southwest China Wildlife Resources Conservation, China West Normal University, Ministry of Education, Nanchong, Sichuan, China; 2 Sericultural Research Institute of Sichuan, Nanchong, Sichuan, China; 3 Manaaki Whenua Landcare Research, Lincoln, New Zealand; 4 Institute of Plant Adaptation and Utilization in Southwest Mountain, China West Normal University, Nanchong, Sichuan, China; Addis Ababa Science and Technology University, ETHIOPIA

## Abstract

Sexual dimorphism is seen in many dioecious plant and animal species, which may influence their trophic interactions. The differences in trophic interactions derived from sexual dimorphism in plants may influence herbivorous performance and population dynamics. Both silkworm (*Bombyx mori* L.) and mulberry (*Morus alba* L.) usually exhibit sexual dimorphism. However, few studies have been conducted on the effect of sex-related silkworm and mulberry pairings on cocoon and silk qualities, which are crucial in sericulture. Here, we compared the differences in cocoon and silk qualities under four feeding combinations (FS-FL: female silkworm fed with leaves from female mulberry trees; MS-FL: male silkworm fed with leaves from female mulberry trees; FS-ML: female silkworm fed with leaves from male mulberry trees; MS-ML: male silkworm fed with leaves from male mulberry trees). The results showed that silkworms exhibited male-biased herbivory with more male mulberry leaves digested. The FS-ML group had higher silk weight and silk ratio of fresh cocoons than the FS-FL group, and the MS-ML group had lower coarse points than the MS-FL group. Compared with groups FS-ML and FS-FL, both MS-FL and MS-ML had smaller cocoons with longer silk lengths and a higher silk ratio of the fresh cocoons. In addition, the Entropy Weight-TOPSIS method showed the cocoon quality rank as FS-ML > FS-FL > MS-FL > MS-ML, whereas silk quality rank was MS-ML > FS-FL > FS-ML > MS-FL. These results indicate that the quality of cocoon and silk is related to the interaction of silkworm and mulberry at the sex level. Furthermore, female silkworms fed with female and male tree leaves have a higher total yield in cocoon production, while male silkworms fed with male tree leaves produced higher silk quality.

## Introduction

As one of the most stable and eco-friendly industries, sericulture has brought great economic benefits to numerous countries globally [1]. Besides traditional uses for all kinds of fabrics, silkworm (*Bombyx mori* L.) silk has been applied in many new areas, such as electronics, medical, and communication [2,3]. According to the International Sericultural Commission report (2018), global total raw silk production was over 177,026 metric tons per year [4]. In 2019, the trade volume of silk products in several major countries and the EU was $28.809 billion, exports were $13.479 billion, and imports were $15.33 billion [5]. Improving cocoon and silk qualities is essential for economic development in the global silk market for several developing countries, e.g., China, India, Uzbekistan, Thailand, Brazil, Vietnam, and Iran [4].

Generally, the qualities of cocoon and silk can be mainly affected by traits of the silkworm, mulberry, or both [6,7]. Previous works have shown that the production and characteristics of cocoon and silk differ among various silkworm strains [8,9]. For example, the hybrid silkworm variety “APM12×APDR105” was superior to 24 other varieties in cocoon weight, silk length and unwinding ratio, with an exception being that strain “APM6×APS12” had a higher cocoon layer percentage [10]. Regarding cocoon dry weight and thickness, silkworm varieties “Baekokjam” and “SK” performed best, but the fiber diameter of “Wonwon” was highest in a trial comparing nine silkworm varieties [8]. Additionally, cocoon and silk production are dependent on the nutritional quality of mulberry leaves. Mulberry variety “Kines” has been found to have higher amounts of protein, nitrogen, and water compared to the mulberry varieties “Ichinose”, “Kenmochi” and “Local”, and the individual and total cocoon weight, cocoon shell weight, and cocoon yield/10,000 per larvae of hybrid “31 × 32” silkworms when reared on “Kines” were higher [1]. Longer silk fiber resulted when silkworm strains were bred on mulberry varieties “Jorhat” and “TR10” compared to “Hmute” and “BC2-59”, likely due to the fact higher amount of calcium, potassium, magnesium and phosphorus in “Jorhat” and “TR10” [6]. However, these studies usually neglected the sexual effect of silkworm and mulberry on the quality of cocoon and silk.

Mulberry (*Morus alba* L.), a dioecious tree species, has been reported sex-specific differences in growth and leaf performance [11–13]. Such as, male mulberry leaves are thicker and have higher relative water content than female leaves [12,13], and female plants suffer more negative effects on morphology, physiology, biomass allocation, or leaf structure than males under stressful habitats [14–16]. In addition, male and female silkworms also exhibit differences in nutrient utilization and silk synthesis, affecting the cocoon size and silk quality [17,18]. Male silkworms tend to produce smaller cocoons with better silk quality [19–21]. These sex-related differences in mulberry leaves and silkworms may influence their trophic interactions. Here, we hypothesized that feeding combinations of a fully factorial combination between two sexes of silkworm and mulberry trees would significantly affect cocoon and silk qualities. To test the hypothesis, the mulberry variety “Yuesang no. 51” and silkworm hybrid variety “871 × 872” were chosen, and the leaf quality of male and female mulberry trees, leaf digestion by male and female silkworms and economic characteristics of cocoon and silk under four sex-related silkworm and mulberry feeding groups were investigated. The results reported here have the potential for practical applicability in the sericulture industry.

## Material and methods

### Plant materials and experimental design

Mulberry “Yuesang no. 51” and silkworm “871 × 872” were collected from the Sericultural Research Institute (30.87N, 106.08E), Sichuan Academy of Agricultural Sciences, in Nanchong, Sichuan Province, China. The dioecious mulberry variety “Yuesang no. 51”, originated from the south of the Yangtze River, has the characteristics of large and thick leaves, high leaf yield, excellent leaf quality, etc. Silkworm hybrid variety “871 × 872”, which has a muscular physique and excellent cocoon silk quality, is the primary silkworm breed in China.

Based on the sexes of the silkworm and mulberry, four feeding combinations were designed (female silkworm fed with leaves from female mulberry trees, FS-FL; male silkworm fed with leaves from female mulberry trees, MS-FL; female silkworm fed with leaves from male mulberry trees, FS-ML; male silkworm fed with leaves from male mulberry trees, MS-ML). Based on the morphological differences between male and female flowers at the flowering period [22], 18 males and 18 females aged three years were selected for the mulberry sex treatment. After the silkworm eggs hatched, larvae were fed with leaves in the rearing room of the mulberry planting base of the Sericulture Research Institute, and the rearing method and environmental conditions followed the industrial standard [6,23]: larvae were fed three times a day (6 h interval) with mature leaves obtained from the top of the mulberry plants. The 1^st^ and 2^nd^ instar silkworms were fed chopped leaves and 3^rd^ to 5^th^ instar silkworms were fed whole leaves. Before the first feed of every instar, the larvae were dusted with bleaching powder (3%). The ambient temperature and humidity were 27±2°C, 80-90% during the 1^st^ to 2^nd^ instar, respectively, and 25±1°C, 65-75% from the third active feeding stage. The leaf quality of male and female leaves and the digestion by male and female silkworms were investigated during this experiment. In addition, traits in cocoon characteristics and silk quality (reflecting the direct economic benefits of sericulture [1,6]) were measured in the Sericulture Machinery Research Laboratory, Sericulture Research Institute. The experiment lasted from May 5 to June 12 (38 days) in 2017.

### Feeding treatment and sex identify of silkworm

**Fig 1:**
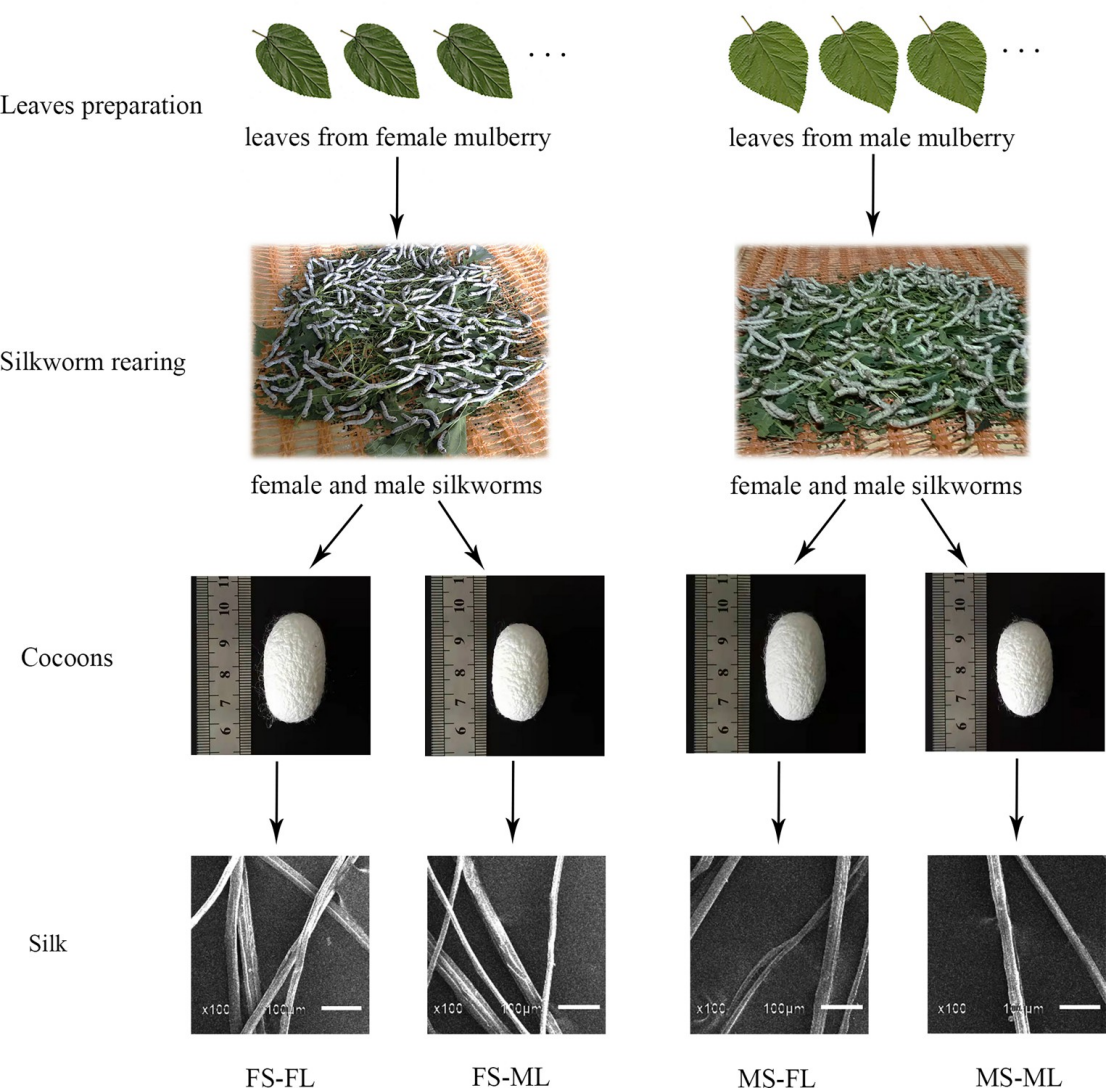
A schematic diagram of silkworms fed with mulberry leaves. FS-FL: Female silkworm fed with leaves from female mulberry trees. MS-FL: Male silkworm fed with leaves from female mulberry trees. FS-ML: Female silkworm fed with leaves from male mulberry trees. MS-ML: Male silkworm fed with leaves from male mulberry trees.

The 1^st^ and 2^nd^ instar larvae were reared together in a frame with leaves that were not separated by mulberry tree sex. At the beginning of the third instar stage, we randomly selected 400 healthy larvae for mulberry sex feeding treatment: 200 larvae were provided with female mulberry leaves and the other 200 larvae were provided with the same amount of male mulberry leaves. Following the larvae spinning their cocoons, 100 cocoons in each of the mulberry sex feeding treatments were selected randomly for cocoon and silk qualities measurement. Silkworm sex treatment under feeding with leaves of each mulberry sex was determined after the fact because the sex of silkworm variety “871×872” could not be distinguished before pupation. By identifying the sex of silkworm pupa inside the cocoons after cocoon silk reeling, we recorded the silkworm numbers of each sex in each of the feeding treatments. Four feeding groups named as FS-FL (female silkworm fed with leaves from female mulberry trees), MS-FL (male silkworm fed with leaves from female mulberry trees), FS-ML (female silkworm fed with leaves from male mulberry trees) or MS-ML (male silkworm fed with leaves from male mulberry trees) (*See* Fig 1). Silk images (x 100) of four groups were obtained using an electronic scanning microscope JSM-6510LV (Jeol Co., Ltd., Japan) (*See* Fig 1).

### Leaf quality measurements of female and male mulberry

Three leaves of LPI (leaf plastochron index; from the first fully expanded leaf on top to bottom) 3-5 of main branches from seven trees in each sex were chosen. Leaf thickness, fresh weight, and leaf area were measured with a thickness gauge (0-12.7 mm, ICE Instrument Co., Ltd., China), electronic balance (MS304TS/02, METTLER TOLEDO Instrument Co., Ltd., Switzerland), and scanner (CanoScan LIDE 210, Canon Inc., Japan), respectively. The average single leaf area was calculated as the ratio of total leaf area by leaf number. After being oven-dried to a constant mass at 105°C for 30 min and 70°C for 48 h, the dry mass was weighed and the water content and leaf mass per area were calculated according to the method of Garnier and Laurent [24]. The dried leaf samples were ground and passed through a 0.25 mm sieve and determined the contents of C and N by an Elemental Analyzer (Vario Macro Cube, Germany). Crude ash, crude fiber, crude fat, and crude protein were measured by referring to the AOAC (the Association of Official Agricultural Chemists) method [25] in the Ecological Technology Corporation, Policn, Lanzhou by a mixed pool sampling method.

### Determination of leaf digestion by silkworm

Fresh leaves were weighed prior to each feeding during the 3^rd^, 4^th^ and 5^th^ instars, and the total fresh weight was summed across the instars. Following feedings, the remaining leaves and silkworm feces were dried to constant weight and recorded, respectively. The leaf digestion by silkworm for each instar from the 3^rd^ to 5^th^ instar according to Rahmathulla et al. [26] was as follows:

$$\mathrm{CW}(g)=\frac{\mathrm{FW}*(1-\mathrm{MC}\%)-\mathrm{RFW}-\mathrm{SFW}}{\mathrm{SN}}$$ (1)

Where CW = dry weight of leaves digestion; FW = fresh weight of feeding leaves; MC = water content (see the previous section); RFW = dry weight of remaining feeding leaves; SFW = dry weight of silkworm feces; SN = silkworm number.

### Cocoon quality estimation

The length, width, volume and weight of cocoon were chosen as critical traits to determine the quality of the cocoons. Cocoon length and cocoon width were detected with a digital caliper with an accuracy of 0.01mm (91511, SATA Co., Ltd., USA). Cocoon weight was determined with an electronic balance (FA2004, Yueping Scientific Instrument Co., Ltd., China) with an accuracy of 0.1 mg. The cocoon volume was measured using a 50 ml cylinder with a drainage method.

### Silk quality measurements

Following methods by Lee [27], we put the cocoons into boiling water to soften the sericin covering around the cocoon fiber. After putting the cooked cocoon into a warm water bath, the cocoon’s groping end was wound onto a fast-turning single grain reeling machine (SD-2008, Sericultural Research Institute, Chinese Academy of Agricultural Sciences) to isolate the silk from the cocoon. During the unravelling process, the number of reeling machine revolutions was recorded. This number was multiplied by a conversion factor of 400 m to 566 machine revolutions to calculate the silk filament length. After the silk was naturally dried, coarse points (the raw silk defects, of which a greater number indicates poorer silk performance) were counted, and the silk was weighed with an electronic balance (FA2004B, Yueping Scientific Instrument Co., Ltd., China) with an accuracy of 0.1 mg. Using the above data, the silk length (SL) and silk ratio of fresh cocoons (SRFC) were calculated according to Kumar et al. [28].

### Evaluation of cocoon and silk qualities based on entropy Weight-TOPSIS method

Entropy Weight-TOPSIS is a comprehensive evaluation method that assigns the weight for each attribute and then evaluates each sample grade by approaching the relative distance of the ideal solution [29]. With the advantages of objectivity and fairness, this method is widely used in various ecological, economic and agricultural studies [30–32]. Cocoon traits (length, width, weight and volume) and silk quality (coarse point, length, weight and silk ratio of fresh cocoons) in four feeding groups were ranked separately by applying this method. We used the calculation process by Sun et al. [30]:

(1) The initial decision matrix

$$A={\left(a_{ij}\right)}_{m\times n}=[\begin{array}{ccc} a_{11} & \cdots & a_{1n}\\ \vdots & \ddots & \vdots \\ a_{m1} & \cdots & a_{mn} \end{array}]$$ (2)

there are *n* evaluation indicators, and each indicator sets have *m* subsets. The evaluation value of indicator *j* in subset *i* is *a_ij_*, which is the average value of each indicator per feeding combination.

(2) Standardization of the decision matrix

If *a_ij_* denotes a benefit, it is as large as possible:

$$r_{ij}^{\prime }=\frac{a_{ij}}{\sqrt{\sum_{i=1}^{m}a_{ij}^{2}}},\;i=1,\;2,\cdots ,\;m;\;j=1,\;2,\cdots ,n$$ (3)

If *a_ij_* denotes a cost, it is as small as possible:

$$r_{ij}^{\prime }=\frac{\underset{i}{\max}(a_{ij})-a_{ij}}{\sqrt{\sum_{i=1}^{m}{\left(\underset{i}{\max}(a_{ij})-a_{ij}\right)}^{2}}},\;i=1,\;2,\cdots ,\;m;\;j=1,\;2,\cdots ,\;n$$ (4)

Then, use the listed formula to make a normalization:

$$R={\left(r_{ij}\right)}_{m\times n}=r_{ij}^{\prime }/\sum_{i=1}^{m}r_{ij}^{\prime },\;i=1,\;2,\ldots ,\;m;\;j=1,\;2,\ldots ,\;n$$ (5)

(3) Determination of the indicators weight

$$e_{j}=-\frac{1}{\ln m}\sum_{i=1}^{m}r_{ij}\ln r_{ij},\;e_{j}\in [0,\;1],\;\mathrm{If}\;r_{ij}=0,\;r_{ij}\ln r_{ij}=0$$ (6)

$$d_{j}=1-e_{j},\;j=1,\;2,\ldots ,\;n$$ (7)

$$w_{j}=d_{j}/\sum_{j=1}^{n}d_{j}$$ (8)

*e_j_* denotes the entropy of indicator *j*, *d_j_* denotes the dispersity of evaluation value of indicator *j*, and the *w_j_* denotes the weight factor. When *r_ij_* is more dispersed, *d_j_* and *w_j_* is larger, indicator *j* is more important.

(4) The weighted matrix of indicators value

The formula calculated by the standardized decision matrix $R={\left(r_{ij}\right)}_{m\times n}$ and entropy weight *w_j_*:

$$v=[\begin{array}{ccc} w_{1}r_{11} & \cdots & w_{n}r_{1n}\\ \vdots & \ddots & \vdots \\ w_{1}r_{m1} & \cdots & w_{n}r_{mn} \end{array}]=[\begin{array}{ccc} v_{11} & \cdots & v_{1n}\\ \vdots & \ddots & \vdots \\ v_{m1} & \cdots & v_{mn} \end{array}]$$ (9)

(5) Determination of the ideal solution

$$V_{j}^{+}=\underset{i}{\max}(v_{ij}),\;i=1,\;2,\cdots ,\;m;\;j=1,\;2,\cdots ,\;n$$ (10)

$$V_{j}^{-}=\underset{i}{\min}(v_{ij}),\;i=1,\;2,\cdots ,\;m;\;j=1,\;2,\cdots ,\;n$$ (11)

*V^+^* denotes the positive ideal solution, *V^-^* denotes the negative ideal solution.

(6) Calculation of the distance and TOPSIS evaluation value

$$D_{i}^{+}=\sqrt{\sum_{j=1}^{n}{\left(v_{ij}-v_{j}^{+}\right)}^{2}},\;i=1,\;2,\cdots ,\;m$$ (12)

$$D_{i}^{-}=\sqrt{\sum_{j=1}^{n}{\left(v_{ij}-v_{j}^{-}\right)}^{2}},\;i=1,\;2,\cdots ,\;m$$ (13)

TOPSIS evaluation value (i.e., relative proximity) is expressed as $D_{i}^{-}/(D_{i}^{+}+D_{i}^{-})$.

*D_i_^+^* denotes the distance between *v_ij_* and positive ideal solutions, *D_i_*^−^ denotes the distance between *v_ij_* and negative ideal solution. The larger the TOPSIS evaluation value, the better the comprehensive performance.

### Statistical analyses

All statistical analyses were conducted with the IBM SPSS Statistics 22.0 (SPSS Inc., Chicago, IL, USA). The independent sample t-test was used to determine the statistical significance of differences in leaf quality between female and male mulberry trees. Within the four feeding groups, one-way ANOVA was used to determine differences in the quality of the cocoon and silk among treatment groups. Two-way ANOVAs were used to evaluate the effects of mulberry sex, silkworm sex, and their combination on cocoon and silk qualities. Differences were considered significant at the *P* < 0.05 level. The Entropy-TOPSIS method was used to evaluate the qualities of the cocoons and silk across the four feeding groups.

## Results

### Differences in the leaf quality of male and female mulberry trees and their effect on the silkworm digestion

**Fig 2:**
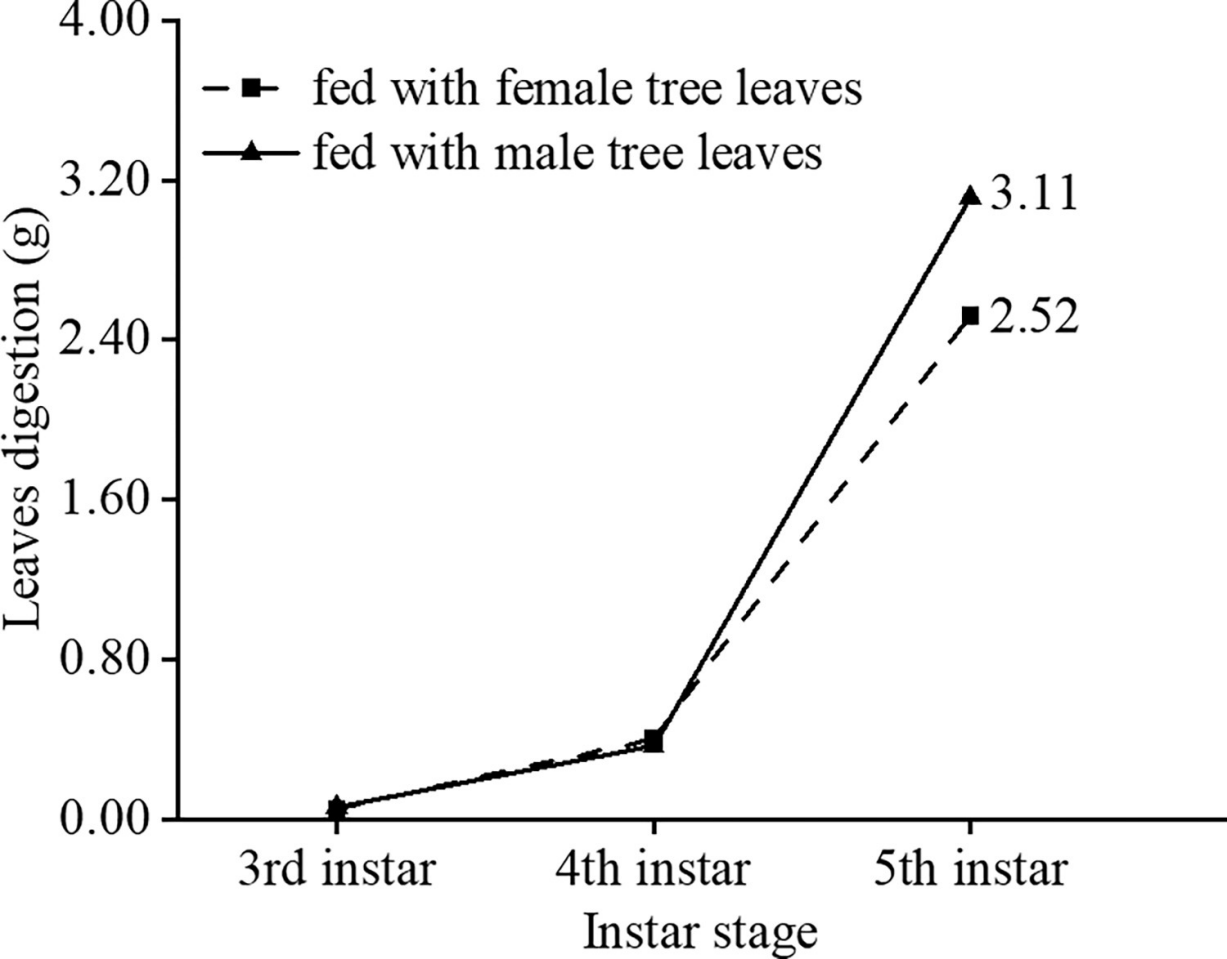
The leaves digestion by silkworm between female and male mulberry tree leaves.

There were significant differences in most leaf traits between male and female mulberry trees (Table 1). Compared with female trees, male trees exhibited higher single leaf area, leaf mass per area, leaf thickness and C/N ratio, but lower content of N, crude protein, and crude ash. However, the contents of water, C, crude fat and crude fiber were similar between the two sexes (Table 1). In the 3^rd^ and 4^th^ instars, leaf digestion of female tree leaves was similar to that of male tree leaves and showed 23.41% higher digestion of male tree leaves than female tree leaves in the 5^th^ instar (Fig 2).

**Table 1:** Differences in leaf quality of leaves from male and female mulberry trees.

Parameter	Female leaves	Male leaves	Significance
Water content (%)	70.19±1.03a	69.05±0.6a	ns
Single leaf area (cm^2^)	99.06±6.17b	129.15±4.24a	**
Leaf mass per area (g/m^2^)	0.55±0.02b	0.62±0.01a	**
Leaf thickness (mm)	0.27±0.01b	0.3±0.01a	**
C (%)	41.86±0.5a	42.08±0.2a	ns
N (%)	2.63±0.12a	1.84±0.11b	***
C/N	16.11±0.82b	23.21±1.1a	***
Crude protein (%)	18.37±0.24a	12.63±0.21b	***
Crude fat (%)	4.29±0.22a	3.91±0.47a	ns
Crude fiber (%)	22.50±1.62a	27.24±1.39a	ns
Crude ash (%)	10.60±0.10a	8.35±0.05b	***

The data show means ± SE (n=7). Different letters in the same row mean significant differences between treatments at 0.05 level. ns, not significant

**, *P* < 0.01

***, *P* < 0.001.

### Effects of sex-related mulberry and silkworm feeding groups on cocoon quality

Significant differences existed in cocoon quality across the four sex-related feeding groups (Table 2). FS-ML treatment had significantly greater values in cocoon length, cocoon width, cocoon weight and cocoon volume than MS-ML, and FS-FL had significantly greater cocoon weight and cocoon volume than MS-FL (Table 2). MS-FL had significantly greater cocoon width and cocoon volume than MS-ML, while having similar cocoon length and cocoon weight (Table 2). In addition, the cocoon traits were significantly affected by silkworm sex, but not the mulberry sex and the interaction of silkworm sex and mulberry sex (Table 2).

**Table 2:** Cocoon quality from each treatment under four sex-related silkworm and mulberry feeding groups.

Treatment	Cocoon number	Cocoon length (mm)	Cocoon width (mm)	Cocoon weight (g)	Cocoon volume (cm^3^)
FS-FL	52	32.97±0.20a	19.07±0.11a	1.85±0.02a	6.86±0.11a
MS-FL	48	32.60±0.21ab	18.82±0.09a	1.51±0.02b	6.51±0.10b
FS-ML	50	32.89±0.20a	19.06±0.13a	1.87±0.02a	6.78±0.12ab
MS-ML	50	32.04±0.21b	18.44±0.11b	1.47±0.01b	6.20±0.10c
*F*_sex1_		ns	ns	ns	ns
*F*_sex2_		**	***	***	***
*F*_sex1×sex2_		ns	ns	ns	ns

FS-FL: Female silkworm fed with leaves from female mulberry trees. MS-FL: Male silkworm fed with leaves from female mulberry trees. FS-ML: Female silkworm fed with leaves from male mulberry trees. MS-ML: Male silkworm fed with leaves from male mulberry trees. The data show means ± SE. Different letters in the same column mean significant differences between treatments at 0.05 level. *F*_sex1_, mulberry sex effect; *F*_sex2_, silkworm sex effect; and *F*_sex1*×*sex2_, interaction effect of mulberry sex and silkworm sex. ns, not significant

**, *P* < 0.01

***, *P* < 0.001.

### Effects of sex-related mulberry and silkworm feeding groups on silk quality

**Fig 3:**
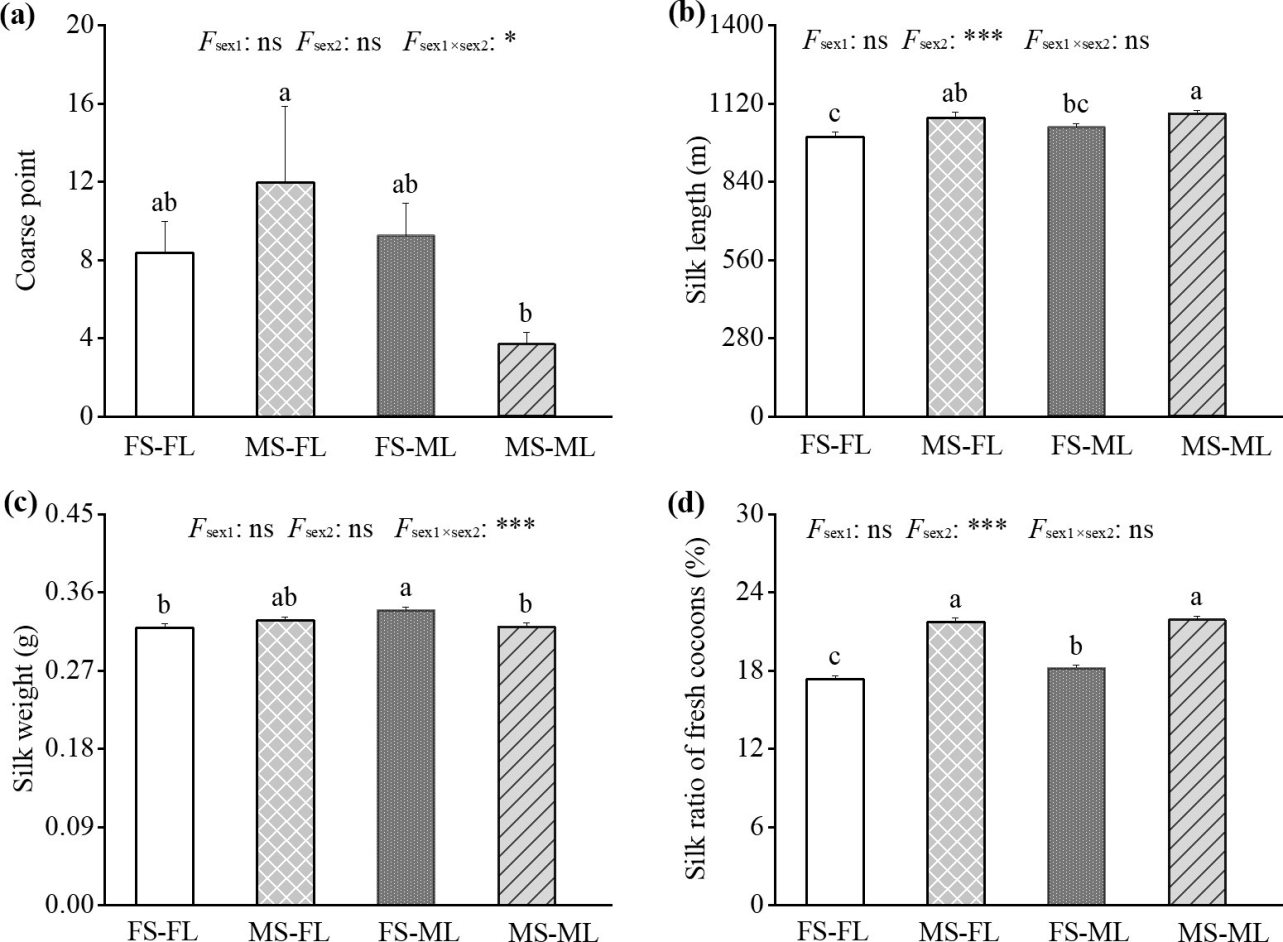
Silk quality from each treatment under four sex-related silkworm and mulberry feeding groups. (a) Coarse point, (b) silk length, (c) silk weight, and (d) silk rate of fresh cocoons. FS-FL: Female silkworm fed with leaves from female mulberry trees. MS-FL: Male silkworm fed with leaves from female mulberry trees. FS-ML: Female silkworm fed with leaves from male mulberry trees. MS-ML: Male silkworm fed with leaves from male mulberry trees. *F*_sex1_, mulberry sex effect; *F*_sex2_, silkworm sex effect; and *F*_sex1*×*sex2_, interaction effect of mulberry sex and silkworm sex. ns, not significant; *, *P* < 0.05; ***, *P* < 0.001.

Significant differences existed in silk quality across the four sex-related feeding groups (Fig 3). MS-ML had significantly greater silk length and silk ratio of fresh cocoons than FS-ML, and MS-FL had significantly greater silk length and silk ratio of fresh cocoons, while lower silk weight than FS-FL. FS-ML had significantly greater silk weight and silk ratio for fresh cocoons than FS-FL, and MS-ML had significantly lower coarse points than MS-FL. In addition, silk length and silk ratio of fresh cocoons were significantly affected by silkworm sex, and coarse points and silk weight were significantly affected by the interaction of mulberry sex and silkworm sex (Fig 3).

### Evaluation of cocoon and silk qualities

The Entropy Weight-TOPSIS method was used to evaluate the cocoon characteristics and silk quality of four sex-related silkworm and mulberry groups separately (Table 3). Across the four groups, the rank of cocoon quality was FS-ML > FS-FL > MS-FL > MS-ML, whereas the class of silk quality was MS-ML > FS-FL > FS-ML > MS-FL. Compared with other groups, FS-FL and FS-ML (female silkworms fed with leaves of female and male mulberry trees) both had larger cocoon shape and weight, and the MS-ML group (male silkworm fed with leaves of male mulberry trees) had the smallest cocoon size and lowest weight but the highest quality silk.

**Table 3:** Evaluation of cocoon and silk qualities with Entropy-TOPSIS method for four sex-related silkworm and mulberry feeding groups.

Treatment	Cocoon quality				Silk quality			
	D^+^	D^-^	D^-^/(D^+^+D^-^)	Class	D^+^	D^-^	D^-^/(D^+^+D^-^)	Class
FS-FL	0.0058	0.0996	0.9446	2	0.4877	0.3762	0.4354	2
MS-FL	0.0940	0.0117	0.1103	3	0.8639	0.0014	0.0017	4
FS-ML	0.0006	0.1055	0.9943	1	0.5767	0.2872	0.3325	3
MS-ML	0.1055	0.0000	0.0000	4	0.0000	0.8639	1.0000	1

FS-FL: Female silkworm fed with leaves from female mulberry trees. MS-FL: Male silkworm fed with leaves from female mulberry trees. FS-ML: Female silkworm fed with leaves from male mulberry trees. MS-ML: Male silkworm fed with leaves from male mulberry trees. D^+^: The distance to the positive ideal solution. D^-^: The distance to the negative ideal solution. D^-^/(D^+^+D^-^): Relative proximity, the final comprehensive evaluation value.

## Discussion

### The effect of leaf quality between mulberry sexes on digestion by silkworms

We found that leaf digestion of female tree leaves was similar to that of male tree leaves in the 3^rd^ and 4^th^ instars, while the 5^th^ instar showed higher digestion of male tree leaves than that of female leaves (Fig 2), which is consistent with the previous study that silkworms were more responsive to feeding at the 5^th^ instar than at other instars [33]. The male-biased herbivory has been related to the sex-related differences in plant leaf quality [34–37]. Our results showed that male leaves contained lower nutrient content (nitrogen, crude protein and ash) but have higher vegetative growth (larger unit leaf area, leaf thickness and leaf mass per area) than female leaves (Table 1). Previous studies have shown that female plants tend to invest more substances, such as nitrogen, phosphorus and carbon, for future reproduction, thus reducing the investment in growth, while male plants have the opposite trade-offs [34,38].

A higher growth rate (more and larger leaves, taller plants, and greater shoot biomass) in males is expected to produce lower leaf secondary defense compounds (phenolics, tannins) [34,38–40], which might result in higher consumption of male plants by herbivores. On the other hand, the higher digestion of male tree leaves may be related to the insect feeding behavior affected by plant leaf nutrients. A study found that *Acronyctodes mexicanaria* caterpillars had a higher relative consumption rate for male tree leaves than female tree leaves of *Buddleja cordata*. This was considered to be due to compensation for the low nutrient content in male tree leaves [41]. In addition, differences in silkworm metabolites, such as sucrose and fructose, affected by feeding different nutrient plants may also cause food intake differences [18]. Overall, these results demonstrate leaf quality differences of a dioecious plant can affect silkworm digestion.

### Effects of sex-related mulberry and silkworm feeding groups on cocoon quality

This study found that silkworm sex significantly affected the cocoon traits, as male silkworms had smaller cocoon shapes and weights (Table 2). Specifically, male silkworms fed with female tree leaves had lower cocoon weight and volume compared to female silkworms fed with female tree leaves, and when fed with male tree leaves, the cocoons of male silkworms had shorter length and width and lower weight and volume compared to cocoons of female silkworms (Table 2). The heavier and larger female cocoons could be caused by female larvae storing more matter as biomass (i.e., pupae) for laying the eggs [42]. Additionally, silkworms obtain nutrients from mulberry leaves [43]; more leaf digestion may benefit the growth and nutrient storage for each sex. However, the cocoon quality showed no significant differences between silkworms feeding on male and female mulberry tree leaves (Table 2). Silkworms fed with male tree leaves had similar cocoon weights as those fed with female tree leaves, despite greater male tree leaf digestion (Table 2), which may be caused by the differences in nutritional efficiency (conversion of food to biomass). Previous work has found that nutritional efficiency is lower when *Acronyctodes mexicanaria* caterpillars fed with male tree leaves of *Buddleja cordata* compared to the nutritional efficiency of feeding on female tree leaves [41]. In addition, we found that male silkworms fed with male tree leaves had the same cocoon weight as those fed with female tree leaves, while the latter had a larger cocoon size (width and volume) (Table 2). This may be due to more coarse points increasing the cocoon’s thickness (Fig 2). Among the four groups, female silkworms fed with female and male tree leaves have the larger cocoon shape and weight, while male silkworms fed with male mulberry leaves had relatively lower cocoon weight and the smallest cocoon size (cocoon width and cocoon volume) (Table 2). Cocoon quality was closely related to sex-related mulberry and silkworm combination feeding groups.

### Effects of sex-related mulberry and silkworm feeding groups on silk quality

Significant differences in silk quality were detected across the four feeding groups. Silk length and silk ratio for fresh cocoons were affected by silkworm sex (Fig 3). Male silkworms fed with male and female tree leaves had longer silk length and a higher silk ratio for fresh cocoons than female silkworms fed with male and female tree leaves, respectively (Fig 3). The higher quality silk of male silkworms compared to female silkworms might be due to male larvae having a higher leaf-silk conversion ratio and not needing to expend energy on egg production and laying [19–21]. In addition, it is worth noting that with the characteristics of finer silk [20], male silkworms fed with male and female tree leaves had longer silk lengths but similar silk weight compared with female silkworms fed with male and female leaves, respectively (Fig 3). On the other hand, we found that both male and female silkworms fed with male tree leaves produced better silk quality than those fed with female tree leaves (Fig 3). For instance, female silkworms fed with male tree leaves had higher silk weight and silk ratio for fresh cocoons, and male silkworms fed with male tree leaves had higher neatness (e.g., fewer coarse points). The result also reflected the interaction of silkworm and mulberry sex on silk quality (coarse point and silk weight). Recent studies showed that silkworms have different gene expression levels in the silk gland across the two sexes [44], and different expression levels of proteins related to silk synthesis after feeding on food with two nutritious levels [45]. Interactions between nutritional levels and animal genes can impact the economically important traits of animals [18,45]. It is thus plausible that the interaction of silkworm genes and mulberry leaf nutrition at the sex level resulted in silk quality inconsistences. However, the underlying mechanism of how the sex-related interaction affects silk quality is unclear and merits future study. These results suggested that male silkworms fed with male tree leaves were more conducive to producing high-quality silk.

### Evaluation of cocoon traits and silk quality under different feeding groups

Previous research has illustrated Entropy Weight-TOPSIS as a reliable method to make decision-making and evaluation [32,46]. In this study, cocoon and silk qualities were evaluated separately by the Entropy Weight-TOPSIS method; based on this method, the cocoon quality rank is FS-ML > FS-FL > MS-FL > MS-ML, and the silk quality rank is MS-ML > FS-FL > FS-ML > MS-FL. The cocoon grade of MS-ML (male silkworm fed with male tree leaves) was the lowest, while this combination produced the highest silk grade (Table 3). This result indicates that large and heavy cocoons do not mean excellent silk quality. However, aside from this male silkworm-male tree leaf group, the cocoon and silk qualities were closely correlated with the other mulberry and silkworm sex groupings.

In this study, the cocoon and silk qualities of silkworms under sex-related mulberry and silkworm feeding groups were comprehensively compared and analyzed, and the corresponding grades were assessed by the Entropy Weight-TOPSIS method. Cocoon production was influenced by silkworm sex but not by the tree sex and sexual interactions. FS-ML and FS-FL groups had higher total yield due to the larger and heavier cocoons of female silkworms (Table 2). However, MS-ML group had relatively better cleanness and longer silk threads (Fig 3). On the one hand, the male silkworms have a higher silk conversion rate than female silkworms. Meanwhile, mulberry sex, silkworm sex, and their interaction also play an important role in improving silk quality, but the specific mechanism needs further study.

Anyway, the sex-related results from our study provide a new insight into sericulture that the silkworm sex, tree sex and their interaction should be considered a factor in improving cocoon production and silk quality. Additionally, as cocoon production and silk quality are not correlated across each sex-related feeding group, there is an opportunity to develop high cocoon production and high silk quality independently to meet the industry’s requirements. Such as, focusing on special breeding technology of female silkworms for high cocoon production or popularizing special breeding technology of male silkworms and cultivating more male mulberry trees in mulberry plantations for high silk quality. Importantly, future work should address the mechanisms controlling the cocoon production and silk quality (e.g., mechanical strength) derived across sex-related feeding groups and investigate whether these sex-related results are consistent across the many varieties of silkworms and mulberry trees.

## Conclusions

This study tested the mulberry leaf quality, leaf digestion by silkworms, and cocoon and silk qualities to explore the effects of sex-related mulberry and silkworm feeding groupings. The results show that the interaction of silkworm and mulberry at the sex level can influence cocoon and silk qualities. Female silkworms fed with female and male tree leaves have the larger cocoon shape and weight, male silkworms fed with male mulberry leaves had the lowest cocoon production but the highest silk quality. Our results, first, find that the cocoon and silk qualities are influenced by the feeding mulberry-silkworm at the sex level, which provides new insights into sericulture development.
